# 39901 Breaking down silos to synergize clinical trial development and initiation: The Clinical Research Support Center, University of Minnesota

**DOI:** 10.1017/cts.2021.677

**Published:** 2021-03-30

**Authors:** Brian L. Odlaug, Francoise Crevel, Nicole Tosun, Ryan Lee, Carrie McKenzie, Melena Bellin, Brenda Prich, Daniel Weisdorf

**Affiliations:** 1 Clinical and Translational Science Institute, University of Minnesota, Minneapolis, MN, USA; 2 Department of Pediatrics and Surgery, University of Minnesota Medical Center, Minneapolis, MN, USA; 3 Division of Hematology, Oncology, and Transplantation, Department of Medicine, University of Minnesota, Minneapolis, MN, USA

## Abstract

ABSTRACT IMPACT: The model of the Clinical Research Support Center at the University of Minnesota of streamlining clinical trial infrastructure can be leveraged by the larger clinical trial community to create valuable efficiencies and facilitate faster initiation of research activities by supporting researchers from concept to dissemination. OBJECTIVES/GOALS: Substantial time, energy, and money are spent bridging disparate resources in research. We describe how the University of Minnesota’s (UMN) Clinical Research Support Center (CRSC) streamlines trial infrastructure, creating valuable efficiencies to support researchers from concept to dissemination. METHODS/STUDY POPULATION: The CRSC, established in 2018 through the Clinical and Translational Science Award (CTSA) program, brings resources together in a single, centralized, and convenient location to help researchers navigate the UMN clinical research startup process and specifically to assist with the development and initiation of a research study from feasibility assessment to project opening. Diverse expertise in components of human subject research is available to support the broad scope of projects at a large institution like the UMN. We present how CRSC services, when coordinated by Clinical Research Specialists, have been used to improve access to clinical research resources during the start up process. RESULTS/ANTICIPATED RESULTS: Since inception in 2018, the CRSC has provided support to over 1700 studies with 437 research projects referred to a Clinical Research Specialist within the CRSC. Of those projects, 97 (22.2%) received comprehensive support from the following expert groups: regulatory guidance (n=74), biostatistics (n=68), clinical (hospital or clinic) partners (n=60), recruitment (n=36), budget development assistance (n=30), and (bio)informatics (n=27). Successful examples of synergies to streamlining study start up include shortening the window between protocol development support from Clinical Research Specialists and IRB submission preparation through to Regulatory Specialists to 3 days. DISCUSSION/SIGNIFICANCE OF FINDINGS: Providing cross-functional support to research teams through the CRSC increases the likelihood of quicker and successful execution and completion of research initiation and subsequently impacts the dissemination of that research to patients and the broader community.

